# Radiofrequency Catheter Ablation-Induced Gastroparesis and Gastrointestinal Distension

**DOI:** 10.5334/jbsr.4226

**Published:** 2026-02-16

**Authors:** Leizhi Ku, Shengpeng Guo, Xiaojing Ma

**Affiliations:** 1 Department of Radiology, Wuhan Asia Heart Hospital, Wuhan University of Science and Technology, No. 753 Jinghan Road, Hankou District, Wuhan 430022, P.R. China; 2 Department of Radiology, Wuhan Asia Heart General Hospital, Wuhan University of Science and Technology, No. 753 Jinghan Road, Hankou District, Wuhan 430022, P.R. China; 3 Department of Echocardiography, Wuhan Asia Heart Hospital, Wuhan University of Science and Technology, Wuhan, 430022, P.R. China

**Keywords:** gastroparesis, gastrointestinal distension, radiofrequency catheter ablation, atrial fibrillation, complication, X-ray iodine contrast radiography, abdominal CT, mosapride citrate

## Abstract

*Teaching point:* The case highlights the importance of identifying gastric complications after RFCA and the need for prompt diagnosis and treatment of gastroparesis with gastrokinetic medication, such as mosapride citrate.

## Case History

**Figure 1:**
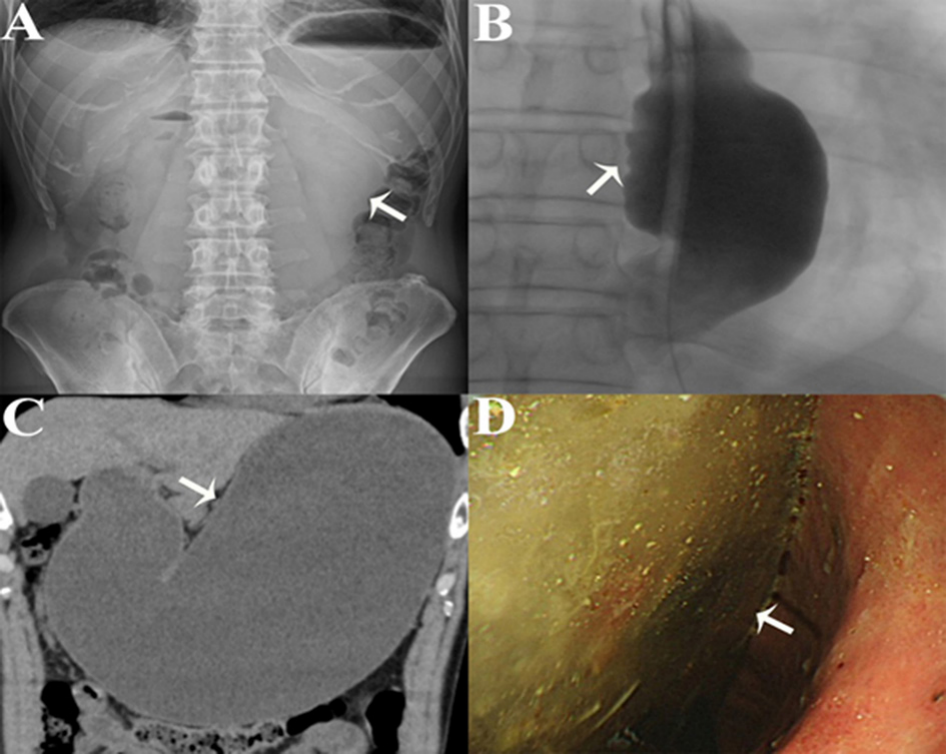
**(A)** An abdominal X-ray suggests gastric distension. **(B)** The X-ray iodine contrast radiography image shows esophageal distension. **(C)** Abdominal CT revealed gastric dilatation without any gastric or intestinal obstruction. **(D)** Esophagogastroduodenoscopy reveals an abundance of food residue, and no obstructing lesion at the pylorus.

A 56-year-old male was referred to the hospital because of vomiting, epigastric pain, and abdominal distension for 5 days. The patient had a medical history of radiofrequency catheter ablation (RFCA) for persistent atrial fibrillation one week earlier. On physical examination, the abdomen was distended but nontender on palpation. Laboratory examinations were unremarkable. An abdominal X-ray suggested significant gastric distension (Figure 1A). Contrast radiography of the upper gastrointestinal tract showed significant esophageal distension (Figure 1B and Video S1). Abdominal CT revealed marked gastric dilatation without gastric or intestinal obstruction (Figure 1C). Esophagogastroduodenoscopy revealed an abundance of food residue (Figure 1D) and no obstructing cause at the pylorus. Gastroparesis and gastrointestinal distension after catheter ablation for atrial fibrillation were diagnosed. After fasting for several days, the patient was subsequently administered mosapride citrate (5 mg, three times a day). His symptoms gradually improved and resolved completely three months after RFCA.

## Comment

Gastroparesis and gastrointestinal distension following RFCA are uncommon extracardiac complications and the most likely mechanism is periesophageal vagal nerve injury. These remain underrecognized complications among gastroenterologists, general physicians, and radiologists, and may be masked by delayed presentation after RFCA. Contrast-enhanced gastric X-ray and plain abdominal CT are essential for accurate diagnosis and timely, appropriate treatment. The characteristic radiographic finding is gastric distension and massive accumulation of food residues [1]. Generally, the management of gastroparesis and gastrointestinal distension is conservative; fasting and bowel rest, gastric decompression, and administration of antiemetics and prokinetic agents are recommended. The case emphasizes the importance of identifying gastric complications after RFC and the need for prompt diagnosis and treatment of gastroparesis with gastrokinetic medication.

## Additional File

The additional file for this article can be found as follows:
